# The Role of Psychological Parental Control and Internalizing Problems in the Etiology of Vigorexia and Orthorexia in Adolescence

**DOI:** 10.3390/children11020259

**Published:** 2024-02-17

**Authors:** Giulia Carlotta Guerra, Antonio Paone, Francesca Lionetti, Maria Spinelli, Mirco Fasolo, Giulio D’Urso

**Affiliations:** University G. D’Annunzio Chieti-Pescara, 66100 Chieti, Italy; giulia.guerra@unich.it (G.C.G.); antonio.paone001@studenti.unich.it (A.P.); francesca.lionetti@unich.it (F.L.); maria.spinelli@unich.it (M.S.); mirco.fasolo@unich.it (M.F.)

**Keywords:** vigorexia, orthorexia, psychological parental control, adolescence, anxiety, depression

## Abstract

The present study aims to explore the forms of psychological parental control that are interconnected with dysfunctional emotional states (i.e., anxiety and depression), and how these internalizing problems may manifest as distorted behaviors (i.e., vigorexic and orthorexic behaviors) during adolescence. Participants included 403 Italian adolescent athletes (231 boys and 172 girls) aged 14 to 18 years. The participants completed self-report questionnaires designed to assess psychological parental control oriented towards dependence and achievement, anxiety and depression, and vigorexia and orthorexia. The results highlight how both forms of psychological parental control predict anxiety and depression. Furthermore, anxiety was found to be linked to both vigorexic and orthorexic behaviors, while depression is connected only to vigorexia. This study delves into the intricacies of parental influence on adolescents, revealing that both dependency-oriented and success-oriented psychological parental control have notable implications for the mental well-being of adolescents. The findings underscore the interconnectedness of these factors, demonstrating that anxiety can set off a chain reaction, leading to engagement in vigorexic and orthorexic behaviors. On the other hand, depression appears to be uniquely associated with vigorexia. These insights contribute to our understanding of the complex dynamics between parental control and adolescent mental health. The implications of this research extend to both theoretical frameworks and practical interventions, emphasizing the need for a nuanced approach to supporting adolescents in navigating these challenges.

## 1. Introduction

Psychological parental control refers to a form of parenting behavior involving the use of psychological or emotional strategies in a coercive way to manipulate adolescent behavior, emotions, and thoughts [1,2,3,4,5,6]. In 2010, Soenens and colleagues [7] introduced two distinct constructs: dependency-oriented psychological control (DPC) and achievement-oriented psychological control (APC). DPC characterizes caregivers who use manipulative and intrusive behaviors to maintain a tight emotional and physical bond with their children. On the other hand, APC refers to encouraging achievement and success with the aim of guiding children’s behavior toward a focus on high standards and accomplishments [7]. Psychological control is different from behavioral control, which focuses on implementing external guidelines and rules and monitoring adolescents’ activities for their well-being [8,9,10,11]. Functional levels of behavior control are linked to reduced substance use, lower levels of delinquency, increased school involvement, and stronger commitment [1,12,13]. It appears that parents employing these strategies may struggle to distinguish their own needs from those of their children [14]. Research on factors linked to psychological control highlights the impact of parents’ internal struggles, particularly in terms of depressive symptoms. Numerous findings indicate that parents, especially mothers, experiencing depression are less sensitive and responsive in interactions with their children [15,16]. Predictably, research has established a connection between mothers’ depressive symptoms and their tendency to exhibit psychologically controlling behavior [15] as a manifestation of parents’ need to maintain a certain level of control within family dynamics [17]. Besides its general negative consequences, psychological control becomes notably problematic during adolescence because it impedes the achievement of typical developmental goals, such as identity formation and autonomy processes, with psychopathological outcomes that may endure into adulthood [6,18,19,20,21].

The literature suggests that parental psychological control, stemming from the anxiety of one or both parental figures, may lead to the development of anxiety symptoms and internalizing behaviors in their child [22,23]. Several studies have revealed a connection between psychological parental control and internalizing symptoms of anxiety and depression [7,24,25,26]. Recently, a different study highlighted the link between inadequate parent–child dynamics and challenges in managing emotions, which may contribute to unhealthy eating behaviors among teenagers, particularly orthorexia among adolescent athletes [27]. Studies show that one of the factors most associated with vigorexia and orthorexia is emotional dysregulation [28,29,30]. However, these recent studies did not take into account the internalizing emotional states (anxiety and depression) of adolescents, which, in line with the literature [7,31], may arise from high levels of parental psychological control and consequently cascade into vigorexic and orthorexic behaviors.

Vigorexia, also known as muscle dysmorphia, is a psychological disorder that affects adolescents and young adults. Individuals with vigorexia have a distorted perception of their body image, always believing that they are not muscular or strong enough. They become obsessed with their appearance and excessively engage in workout routines and weightlifting and consuming high-protein diets. This disorder can have severe consequences on physical health, such as muscle strains, joint injuries, and malnutrition. Vigorexia can result from obsessive behaviors, but it can also be linked to symptoms of body dysmorphic disorder [29]. The presumed motivational factors influencing both the drive for muscularity and body preoccupation revolve around varying levels of anxiety. If individuals are concerned about their physical appearance or have obsessive thoughts about their appearance, they are likely to exhibit elevated levels of body focus. Given that the societal ideal for the male physique leans towards hypermesomorphism [32], cultivating a drive for muscularity is a viable strategy for alleviating body-related anxiety. Furthermore, trait anxiety showed a significant negative correlation with body image; in other words, elevated trait anxiety reliably predicted a less favorable body image [29]. Vigorexia has also been found to be positively associated with anxiety symptoms [33] and symptoms related to depression [30,33].

Orthorexia, on the other hand, involves an obsessive focus on consuming healthy food. Adolescents with orthorexia become excessively concerned about the quality and purity of their food, often avoiding certain food groups they perceive as “unhealthy” or “unclean”. This obsession with healthy eating can lead to significant weight loss, nutrient deficiencies, and social isolation. Orthorexia is not formally acknowledged as an eating disorder or obsessive–compulsive disorder in either the DSM-5 or ICD-10, but suggested diagnostic guidelines have been issued. Over recent years, research in the field has concentrated on refining diagnostic standards, gauging prevalence rates, and exploring psychosocial factors linked to the condition [34]. Self-reported experiences of depression, negative mood, thoughts of self-harm, anxiety, body image concerns, and a diagnosis of major depressive disorder all show positive correlations with orthorexia nervosa symptoms [28,35]. Orthorexic behavior, characterized by an obsessive focus on healthy eating, can lead to compulsive eating and contribute to social isolation. This is consistent with behaviors commonly associated with depression, where symptoms can manifest as a loss of interest in almost all activities [28]. Additionally, a link between anxiety and orthorexic behaviors has been established. Psychological apprehension and distress are intricately linked to the adoption of restrictive eating habits, as demonstrated by the heightened levels of physical- and health-related apprehension observed in those with orthorexia nervosa (ON) [28]. This association is intuitive, given that orthorexia entails an obsessive fixation on consuming exclusively healthy foods, thereby amplifying anxiety levels in individuals grappling with ON. Conversely, restrictive eating patterns like orthorexia might also stem from anxieties surrounding health worries, and the perceived adverse outcomes linked to an imperfect diet or the ingestion of perceived undesirable foods [28]. Considering these theoretical premises, the present study aims to contribute to the existing literature by exploring the following research question (RQ): which forms of psychological parental control are interconnected with dysfunctional emotional states (i.e., anxiety and depression), and how do these emotional states translate into distorted behaviors (i.e., vigorexic and orthorexic behaviors) during adolescence? Consistent with the developmental cascade perspective, dysfunctional family legacies may carry debris that can manifest in internalizing behaviors, which, in turn, can transform into dysfunctional behaviors characteristic of emerging dependencies. In other words, according to this perspective, initial disruptions or changes in one domain, such as family dynamics, can trigger a cascade of effects that reverberate through various aspects of an individual’s development, influencing subsequent behaviors, experiences, and outcomes.

## 2. The Current Study

### 2.1. Method

#### Participants and Procedure

Participants in this study included 403 Italian adolescent athletes (231 boys and 172 girls) aged 14 to 18 years (M = 16.44; SD = 0.83). The participants were involved in competitive activities, such as football and basketball, and/or consistent sports-related activities. All participants self-identified as athletes. The majority of the sample participants came from central Italy (60%), 17% came from the north of Italy, and 23% came from the south of Italy. Most of them attended vocational schools (63%). Informed consent was also obtained from both the parents and adolescents. The data collection took place in 2019, specifically between October and December. The adolescents filled out self-report assessments concerning muscle dysmorphia, psychological parental oversight, and pathological concern during classroom sessions, under the guidance of the researchers. All protocols involving human subjects adhered strictly to the ethical guidelines set forth by the institutional and/or national research committee, aligning with the principles outlined in the 1964 Helsinki Declaration and its subsequent revisions or equivalent ethical standards.

### 2.2. Measures

*Vigorexia or Muscle Dysmorphia*. We used the Muscle Dysmorphia Disorder Inventory (MDDI; [36]), a questionnaire comprising 13 items designed to assess symptoms of muscle dysmorphia. Responses to items on the MDDI are recorded using a 5-point Likert scale, ranging from 1 (never) to 5 (always). The MDDI evaluates three primary facets of muscle dysmorphia: Drive for Size, Appearance Impairment, and Functional Impairment. Examples of items include statements like ‘I perceive my legs as being too thin’, ‘I believe I possess excessive body fat’, and ‘I experience anxiety when I miss one or more workout sessions’. In this study, we computed a composite muscle dysmorphia score (α = 0.78 vs. 0.82) to gain comprehensive insight into the atypical mindset, considering all dimensions collectively. The total score is calculated by summing the scores of the subscales, with a suggested threshold of >39 points indicating problematic symptoms. Total scores range from 13 to 65, with scores surpassing 39 being indicative of addictive or obsessive tendencies. In our investigation, the scores ranged from 14 to 40 (mean = 36.5; standard deviation = 10.3). However, to normalize frequency distributions, we standardized the scores using the mean value scale.

#### 2.2.1. Orthorexia

The ORTO-15 questionnaire in its Italian version [37] was employed to evaluate tendencies toward orthorexia. This questionnaire comprises 15 statements and utilizes a 4-point Likert scale, ranging from 1 (never) to 4 (always). Collectively, the statements gauge the manifestation of obsessive behaviors in individuals’ selection, acquisition, preparation, and intake of foods deemed healthy (for instance, ‘Do you make food choices based on your health concerns?’) (for the current study, α was 0.91 vs. 0.86).

*Psychological Parental Control*. The Italian adaptation [38] of the Dependency-Oriented and Achievement-Oriented Parental Psychological Control Scale (DAPCS; [7]) was utilized to evaluate adolescents’ perceptions regarding their parents’ psychological influence. This scale consists of 16 items examining dependency-oriented psychological control (DPC; for example, ‘My father/My mother is only warm towards me if I depend on him/her rather than on my friends’; α = 0.83) and achievement-oriented psychological control (APC; for instance, ‘My father/My mother makes me feel guilty if my performance is subpar’; α = 0.81). The reliability of the original version of the DPC scale was assessed using Cronbach’s alpha, resulting in 0.86 for maternal assessments and 0.83 for paternal assessments, whereas for the APC scale, Cronbach’s alpha yielded 0.93 for maternal assessments and 0.91 for paternal assessments. Respondents indicated their agreement using a 5-point Likert scale, ranging from 1 (strongly disagree) to 5 (strongly agree). Higher scores on both scales are indicative of increased levels of psychological control exerted by parents [38].

#### 2.2.2. Anxiety and Depression

We administered the Italian version of the Depression Anxiety Stress Scale (DASS-21; [39]), a 21-item measure composed of three self-report scales aimed to assess the core symptoms of depression, anxiety, and stress, since the literature has highlighted the link between self-reported depression and anxiety symptoms with ON symptoms. Each scale includes seven items divided into subscales, i.e., items 3, 5, 10, 13, 16, 17, and 21 constitute the depression subscale (e.g., ‘In the last 7 days, I couldn’t seem to experience any positive feeling at all’; ‘In the last 7 days, I found it difficult to work up the initiative to do things’); items 2, 4, 7, 9, 15, 19, and 20 constitute the anxiety subscale (e.g., ‘In the last 7 days, I experienced trembling’; ‘In the last 7 days, I was worried about situations in which I might panic and make a fool of myself’); and items 1, 6, 8, 11, 12, 14, and 18 constitute the stress subscale (e.g., ‘In the last 7 days, I tended to over-react to situations’; ‘In the last 7 days, I felt that I was using a lot of nervous energy’). Participants responded using a 4-point Likert scale varying from 0 (never) to 3 (almost always). For this study, we used the mean score of the anxiety (α = 0.86) and depression scales (α = 0.87) only. The measures of the original version are α = 0.81 for depression and α = 0.73 for anxiety.

## 3. Analysis Plan

To explore our research question, we conducted a path analysis using MPlus. The model aimed to investigate whether dependence-oriented and achievement-oriented parental psychological control were linked to anxiety and depression. Simultaneously, the model examined whether anxiety and depression, in turn, predicted vigorexic and orthorexic behaviors. In the model (the model was tested on both genders as no significant differences were observed between males and females; in other words, when testing separate models for males and females, the effects were found to be equivalent), we also incorporated the correlations between dependence-oriented parental psychological control and achievement-oriented parental psychological control, anxiety and depression, and vigorexia and orthorexia.

## 4. Results

**Figure 1:**
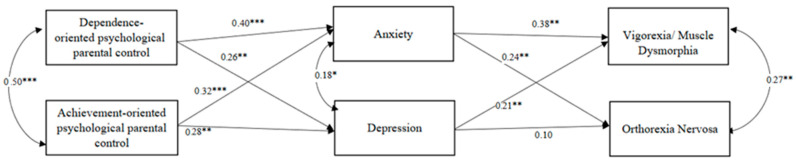
Model summary. Note: pathways with STDYX standardized estimates are displayed. * *p* < 0.05, ** *p* < 0.01, *** *p* < 0.001. R^2^ Anxiety = 0.36. R^2^ Depression = 0.29. R^2^ Vigorexia = 0.30. R^2^ Orthorexia = 0.18.

Model fit statistics indicate that the path analysis model fits the data well, with a root mean square error of approximation (RMSEA) of 0.2 and a comparative fit index (CFI) of 0.97. The model reveals that dependency-oriented parental psychological control positively predicts depression (b = 0.26; *p* < 0.001) and anxiety (b = 0.40; *p* < 0.001); achievement-oriented parental psychological control also positively predicts depression (b = 0.28; *p* < 0.001) and anxiety (b = 0.32; *p* < 0.001). Moreover, depression positively predicts vigorexia (b = 0.21; *p* < 0.001) but does not predict orthorexia. On the other hand, anxiety positively predicts both vigorexia (b = 0.38; *p* < 0.001) and orthorexia (b = 0.24; *p* < 0.001). The model is shown in Figure 1.

## 5. Discussion

The study aimed to investigate, within a group of adolescent athletes, whether parentally oriented psychological control towards dependence and success was associated with anxiety and depression. Moreover, this research explored whether these internalizing behaviors could cascade into, or somehow be connected to, exercise addiction (vigorexia) and orthorexia. Consistent with the risk factors paradigm [40], we sought to examine whether parental influences impact dysfunctional emotional states and whether these states can transform into atypical patterns of dependence and/or obsession. In particular, the results highlight that parentally oriented psychological control towards dependence and success is associated with anxiety and depression. This could be justified by the fact that when parents employ these psychological control practices, there is a significant increase in the levels of anxiety and depression in their children. Put differently, excessive reliance on psychological control within the familial setting could correlate with adverse effects on the mental well-being of adolescents, as evidenced by heightened symptoms of anxiety and depression [4,7].

The imposition of rigid standards and expectations, particularly those linked to success, may create a sense of inadequacy and a fear of failure, fostering an environment conducive to anxiety and depression. Recognizing the significance of comprehending family dynamics, especially the influence of parental caregiving methods on the mental health of adolescents, underscores the need for nurturing and balanced parenting strategies to enhance children’s emotional welfare. Additionally, considering the autonomy development process, adolescents strive to establish a sense of independence and identity. Parental control that leans towards dependence may impede this process, leading to internal conflicts and emotional distress [6,21]. The adolescents may experience pressure to conform to parental expectations, which can contribute to a compromised sense of self and emotional difficulties [20,41]. Furthermore, the results resonate with cognitive–behavioral perspectives, where negative thought patterns and beliefs influenced by parental control can contribute to the maintenance of anxiety and depression [7,24,25,42]. The constant reinforcement of dependence and success as primary indicators of worth may shape distorted cognitions, reinforcing negative emotions [3].

At the same time, depression is associated with vigorexia but not with orthorexia. Firstly, considering the motivational aspects of exercise addiction, individuals with depression may be more prone to engage in excessive physical activity as a coping mechanism. Exercise, in this context, might serve to alleviate depressive symptoms or provide a temporary escape from negative emotions. This aligns with the self-medication hypothesis [43], suggesting that adolescents with depression can turn to certain behaviors, such as excessive exercise, to regulate their emotional state [4]. Instead, the lack of connection with orthorexia could be interpreted through a different lens. Orthorexia, characterized by an obsessive focus on healthy eating, might not be directly linked to depressive tendencies. Alternatively, it could be influenced by different psychological factors, such as anxiety related to the desire for control over one’s dietary intake. Indeed, our study suggests how anxiety is not only associated with orthorexia but also vigorexia. From a developmental perspective, anxiety during adolescence can be a normative response to the challenges and uncertainties associated with this life stage [44]. Adolescents may experience heightened social pressures, academic demands, and identity exploration, contributing to increased stress levels. Engaging in excessive exercise or following rigid diets might serve as maladaptive coping mechanisms to manage or alleviate anxiety. In this context, the need for control over one’s body and the pursuit of an idealized physique may provide a sense of structure and predictability, serving as a compensatory strategy for managing internal emotional turmoil [45]. In line with the cognitive–behavioral model [46], distorted cognitions about body image and the self play a crucial role in the etiology and maintenance of vigorexia and orthorexia. Adolescents with anxiety may harbor irrational beliefs about their appearance, contributing to a perpetual cycle of compulsive exercise as a means of achieving an elusive and idealized body image or by seeking out a perfect diet. The pressure to conform to societal standards of beauty, especially heightened during adolescence, can intensify these irrational beliefs. Consequently, adolescents may be particularly vulnerable to developing maladaptive behaviors, such as excessive exercise or strict dieting, in an attempt to cope with the anxieties associated with body image concerns. Moreover, the reinforcement of exercise and the focus on a highly nutritious diet to cope with anxiety can perpetuate maladaptive behaviors, creating a self-reinforcing loop.

In particular, when anxiety or depression stems from pressure exerted by forms of parental psychological control, it can frustrate the adolescent to such an extent, that because they never feel up to parental expectations or able to be autonomous, they channel their internalizing problems into a disengaged behavior that provides gratification [28,29,31,47]. Aligned with previous research, experiencing psychological control from their parents may prompt adolescents to adopt dysfunctional strategies as a means of emancipating themselves from parental authority [47]. The way parents respond to their child’s emotions plays a significant role in shaping adolescents’ ability to regulate their own emotions. When parents respond inappropriately or inconsistently, adolescents may develop unhealthy coping strategies, which can transform into both internalizing and externalizing behaviors. Essentially, the emotional dynamics between parents and children have a profound impact on adolescents’ emotional regulation and behavioral tendencies. Emotion socialization refers to the processes through which parents teach their children about emotions, including how to recognize, express, and regulate them effectively. Parental responses to their child’s emotions serve as important models for how children learn to manage their own emotions. For instance, if a parent consistently validates and supports their child’s emotions, the child is more likely to develop healthy emotion regulation skills. Conversely, if a parent dismisses or invalidates their child’s emotions, the child may struggle with regulating their emotions and may be more prone to behavioral problems (e.g., anxiety and depression). Adolescents who receive consistent and supportive responses from their parents are more likely to develop adaptive coping mechanisms and exhibit fewer behavioral problems compared to those who experience inconsistent or negative responses from their parents [48].

The study results must also be considered in light of the limitations they present. Firstly, relying solely on self-report questionnaires may introduce potential biases, as adolescents might tend to minimize or exaggerate perceptions about themselves and their parental relationships, thereby affecting the accuracy of the reported data. To address this limitation, future studies should incorporate data from additional sources, such as parents, and employ a combination of quantitative and qualitative methods. This may include interviews, observational studies, and other implicit tools to provide a more comprehensive understanding and enhance the validity of the findings. Furthermore, the cross-sectional nature of the study prevents the establishment of causality between variables. Additionally, the method of data collection utilized in our study involved gathering data at a single time point and focused solely on a sample of Italian adolescent athletes. This may limit the generalizability of our findings, given the specific cultural background of our sample. To address this, it is recommended that the same model be tested across different samples and developmental stages to improve the external validity of the findings and enhance the robustness of the conclusions. Moreover, future research should consider longitudinal designs to better capture the dynamic interplay between variables over time and provide stronger evidence for causal relationships. Additionally, exploring the effect of protective factors, such as peer support, on the proposed model could enhance the depth of analysis. A significant limitation of this study is the lack of data from a clinical sample. This decision was made for ethical reasons. Consequently, the results of this study may not be directly generalizable to the clinical population. However, strict selection criteria were adopted to ensure the validity and reliability of the data collected by other available means. Another notable limitation of this study is the overrepresentation of male participants, potentially restricting the applicability of the findings to diverse gender identities. Future investigations should prioritize a more inclusive sample to explore potential gender-related distinctions and subtleties within the results more comprehensively. Finally, the gender of the adolescent athlete, the socio-economic status of the family, and the level of pressure exerted by the coach were not considered in the results. Therefore, future studies could take these aspects into account.

## 6. Conclusions

This study can improve our understanding of how different forms of parental psychological control can affect adolescent developmental outcomes. It is important to promote more functional parental strategies, such as encouraging communication, spending quality time together, providing emotional support during difficult times, guiding adolescents in conflict resolution and problem-solving, and actively listening to adolescents’ concerns and perspectives. These practices should encompass the implementation of evidence-based strategies aimed at fostering healthy family dynamics. Among these strategies is the promotion of open and transparent communication within the family unit, which encourages the sharing of thoughts, feelings, and experiences among family members. Research suggests that such communication patterns facilitate emotional expression, enhance understanding, and promote the development of adaptive coping mechanisms in adolescents. Furthermore, allocating quality time for shared activities and meaningful dialogue is essential in cultivating familial closeness and reinforcing interpersonal bonds. Studies indicate that regular engagement in positive family interactions correlates with higher levels of emotional well-being and overall family cohesion. These shared experiences not only create lasting memories but also serve as opportunities for parents to model effective communication skills and emotional regulation strategies. Moreover, providing consistent emotional support during challenging circumstances plays a pivotal role in adolescents’ emotional resilience and psychological adjustment. The scientific literature underscores the importance of parental warmth, empathy, and validation in buffering against the negative impact of stressors on adolescent mental health. Adolescents who perceive having greater levels of parental support are more likely to exhibit adaptive coping responses and exhibit fewer symptoms of anxiety and depression. Additionally, guiding adolescents in constructive conflict resolution and problem-solving techniques equips them with invaluable skills for navigating interpersonal challenges and managing emotional arousal effectively. Teaching adolescents how to identify and regulate their emotions, negotiate conflicts assertively, and generate adaptive solutions fosters emotional intelligence and promotes prosocial behavior. Empirical research suggests that adolescents who receive training in these areas demonstrate higher levels of social competence, emotional self-efficacy, and conflict resolution skills, which are crucial components of emotional and social maturity. In this vein, the study underscores the critical importance of fostering autonomy and individuality in adolescents to safeguard the development of crucial emotional and relational skills, thereby mitigating the risk of maladaptive behaviors in the long term. Orthorexic and vigorexic behaviors, if left unchecked and unmanaged at the level of individual identity, have the potential to compromise social and relational domains, impeding the successful completion of developmental tasks. Central to this endeavor is the promotion of positive parenting practices that prioritize attentive listening to the needs of adolescents. By nurturing an environment of open communication and empathetic understanding, parents can play a pivotal role in supporting adolescents’ emotional and behavioral regulation processes. By doing so, adolescents are empowered to make conscious choices and decisions guided by their intrinsic needs, rather than resorting to maladaptive behaviors as a means to escape perceived forms of parental psychological control. Furthermore, it is imperative to recognize that behaviors manifesting from deeper emotional roots are often indicative of unmet needs or underlying psychological distress. By addressing these underlying emotional factors through empathetic support and guidance, parents can help adolescents cultivate healthier coping mechanisms and adaptive responses to challenges. This proactive approach not only fosters emotional resilience but also strengthens the parent–child bond, laying a solid foundation for adolescents to navigate the complexities of adolescence with confidence and self-assurance.

## Data Availability

The data presented in this study are available on request from the corresponding author. The data are not publicly available due to privacy and ethical.
